# Urine washing and urinary odour profiles in relation to dominance rank status in wild male capuchin monkeys (*Cebus imitator*)

**DOI:** 10.1098/rsos.250608

**Published:** 2025-07-16

**Authors:** Alice C. Poirier, Nelle K. Kulick, Suheidy Romero Morales, Marlen Kücklich, Brigitte M. Weiß, Claudia Birkemeyer, Anja Widdig, Amanda D. Melin, Katharine M. Jack

**Affiliations:** ^1^ Department of Anthropology and Archaeology, University of Calgary, Calgary, Alberta, Canada; ^2^ Department of Anthropology, Tulane University, New Orleans, LA, USA; ^3^ Área de Conservación Guanacaste, La Cruz, Guanacaste, Costa Rica; ^4^ Behavioural Ecology Research Group, Faculty of Life Sciences, Institute of Biology, University of Leipzig, Leipzig, Germany; ^5^ Department of Human Behavior, Ecology and Culture, Max-Planck Institute for Evolutionary Anthropology, Leipzig, Germany; ^6^ Mass Spectrometry Research Group, Institute of Analytical Chemistry, University of Leipzig, Leipzig, Germany; ^7^ German Centre for Integrative Biodiversity Research (iDiv) Halle-Jena-Leipzig, Leipzig, Germany; ^8^ Department of Medical Genetics, University of Calgary, Calgary, Alberta, Canada; ^9^ Alberta Children’s Hospital Research Institute, Calgary, Alberta, Canada

**Keywords:** urine washing, olfactory communication, volatile organic compounds, chemosignalling, male dominance rank status, alternative male morphologies, primates

## Abstract

Urine plays an essential role in mammalian olfactory communication, although its potential role in primates has long been overlooked owing to focus on their visual adaptations for communication. Here, we combined behavioural and chemical data to test the role of urine in signalling male dominance in white-faced capuchins (*Cebus imitator*). We predicted that: (i) urine washing (i.e. depositing urine onto hands/feet and rubbing them onto substrates) is more frequently performed by alpha than subordinate males; and (ii) the chemical composition of alpha male urine is distinct from that of subordinates. We collected 457 h of focal behavioural follows and 153 urine samples from 24 males in five groups at Sector Santa Rosa, Área de Conservación Guanacaste, Costa Rica. We extracted urinary volatile compounds into thermal desorption tubes and analysed them by gas chromatography-mass spectrometry. We found that alphas urine wash more than subordinates, especially during the dry season when urinary odours can last longer and intergroup interactions are more frequent. Additionally, dominance rank predicted a modest fraction of overall sample chemical dissimilarity. Our results support the hypothesis that urine may be an olfactory signaling medium; future experimental research is needed to test the extent to which urinary odours may be cues versus evolved signals.

## 1. Introduction

Olfaction is a widespread and common form of communication in mammals, including primates. Odourant chemicals can be deposited in the environment through a variety of mechanisms, including excretion in the form of urine and faeces, or secretion from specialized skin glands, after which they can dissipate into the environment or be deposited onto body parts or substrates [1,2]. The odourants released from excretions and secretions belong to a large array of chemical groups and exhibit varying volatility (reviewed in [2,3]). They range from very small compounds of high volatility that can travel long distances in air or water, to non-volatile compounds that may be retained on the substrate for extended periods. Such versatility in chemical cues and signals allows for the existence of complex mechanisms of olfactory communication adapted to the animals’ biology, behaviour and ecology [1,4]. The characterization of biologically active olfactory signals and cues involved is still limited, in part owing to the chemical complexity of the odourant compounds involved in animal communication [5], and the methodological difficulties of recording and quantifying odour cues, especially in field conditions [6]. Commonly used methods include the collection of odours through swabs rubbed on the scent gland or body (e.g. antebrachial and brachial gland secretions of captive ring-tailed lemurs, *Lemur catta* [7]; suprapubic gland secretions of wild tamarins, *Saguinus imperator* and *Leontocebus weddelli* [8], scent traps such as thermal desorption tubes (e.g. body odour of Barbary macaques, *Macaca sylvanus* [9]) or directly into a container (e.g. mouse urine [10]) and subsequently transported to a laboratory for analysis by gas chromatography (GC; for volatile compounds) or liquid chromatography (for semi- and non-volatile compounds) linked to mass spectrometry (MS).

As in other mammalian taxa, primate olfactory communication has been shown to function in advertising territorial boundaries, individual identity, health, reproductive status and/or dominance rank status [9,11–21]. The complex olfactory repertoire of primates is central to many aspects of their sociality [22,23]. A number of taxa, including callitrichids [24] and lemurs [25], possess highly specialized scent glands which they use to deposit scent-marks in their environment. Amongst catarrhine primates, mandrills, *Mandrillus sphinx*, signal dominance and individuality through their sternal secretions [26]. Olfactory communication requires not only signal generation, but also perception by receivers. In many mammals, the vomeronasal organ is involved in the reception of chemical signals and cues [27,28]. Mandrills display Flehmen behaviour–consisting of lip curling, head raising and a long inhalation with the nostrils usually closed, which facilitates the transfer of volatile chemicals into the vomeronasal organ inside the mouth [29,30]. Yet, there is still a debate over the functionality of the vomeronasal organ in catarrhine primates and apes [31–33].

Containing a mixture of metabolic products, urine excretion is a process that has been co-opted as a signalling pathway in many mammals [34]. Yet, the extent to which urinary odours may be cues (i.e. informative byproducts) versus evolved signals (i.e. shaped by natural selection) has not been clearly established for many species [35–37]. Urine has been shown to be chemically informative regarding the individual’s species [38,39], group membership [40,41], identity [42,43], sex [44,45], age [46,47], dominance rank status [48], health status [49,50], reproductive state [51,52] and mate quality [53,54]. In the context of olfactory communication, urine deposition is sometimes performed at specific locations in the territory [55] or on specific substrates [56]. Urine marking may also be executed in the presence of certain conspecifics [54,57], and associated with visually conspicuous behaviours [58,59]. One such behaviour is urine washing (UW), in which animals deposit urine on their palms or feet and rub it onto substrates in the environment, their fur or conspecifics [60]. UW by primates has notably been reported in a number of platyrrhine (i.e. Central and South American monkeys [61–69]) and strepsirrhine (i.e. lemurs, galagos, pottos and lorises [39,58,70,71]) species. The broad distribution of this behaviour across these primate taxa, both of which are known to rely heavily on olfactory communication [23,72], suggests a link between this behaviour and communication via urinary volatile compounds.

In this study, we examine the olfactory-associated behaviours, specifically UW and the chemical odour profiles of male white-faced capuchins (*Cebus imitator*), as a potential mechanism for communicating male dominance rank status. This species is characterized by alternative male morphology, with an ‘alpha morph’ corresponding to the male of highest dominance rank, and a ‘subordinate morph’ corresponding to the other adult males in the group [73,74]. Although capuchins (*Cebus* and *Sapajus* spp.) lack obvious, external scent glands, their olfactory repertoire appears substantial and they are thought to communicate extensively through odours [75]. Previous studies show that capuchins perform olfactory-related behaviours, for example, sniffing and licking the body and the excretions of themselves and conspecifics [61,66,68,76]. Capuchins also perform UW (electronic supplementary material, video S1), for which several functions have been proposed, including intraspecific olfactory communication, but also non-communicatory functions such as thermoregulation, spatial orientation, improved mobility through improved grip on substrates and protection against parasites (last reviewed in [77]). For instance, behavioural studies on captive tufted capuchins (*Sapajus apella*) support a thermoregulatory function of UW, with higher rates of this behaviour happening in hotter and/or drier environmental conditions [66,78,79]. Another set of behavioural studies seems to indicate that UW would mainly serve a function in intraspecific olfactory communication in capuchins [68,80,81], and thus could potentially be linked to the condition-dependent (i.e. dominance rank-based) expression of alternative male morphology.

After attaining alpha status, capuchin males experience a dramatic increase in androgen levels and display enlarged secondary sexual characteristics, such as exaggerated jaw lines and brow ridges, and increased piloerection [73,74,82–85], which differentiate them from the other males in the group. While we still know little about the proximate triggers leading to these morphological and physiological differences, there is some evidence in rodents [86–88], reptiles [89] and fishes [90] and in strepsirrhine primates (i.e. grey mouse lemurs, *Microcebus murinus* [91]) that volatile odours emitted by dominants (e.g. in urine or from scent glands) may suppress androgen production in subordinates and, possibly, their ability to develop the full suite of secondary sexual traits. The extent to which this mechanism exists has not yet been investigated in male capuchins.

Based on these findings and our knowledge of dominance in the study species, we predicted that: (i) *UW is more frequent in alpha than subordinate males*, supporting the hypothesis that UW plays a role in the intraspecific communication of dominance rank status in male white-faced capuchins; and (ii) *the urinary chemical profiles of alpha males are distinct from those of subordinate males*, which would support the hypothesis of a dominance-specific urinary odour composition that may play a role in the maintenance of dominance rank status in this species. Specifically, we predicted that male dominance rank would be a predictor of the chemical composition of urinary odours, by affecting (iia) the entire chemical profile, and/or (iib) certain specific urinary volatile compounds. Previous research has shown that mammalian urine composition can vary with demographic variables [2,27,92]; therefore, although not the main focus of this study, we additionally investigated the influence of male age and group ID on UW behaviour and urine chemical composition. Moreover, as previous research on the same study species has evidenced relationships between male UW behaviour and seasonality [77,93–95], we also assessed the influence of environmental conditions on UW behaviour and on the chemical composition of male capuchin urine. We predicted that these demographic and environmental variables would have a significant effect on urinary chemical composition, albeit lower than that of male dominance rank status.

## 2. Methods

### 2.1. Study species and site

White-faced capuchins, *C. imitator*, are medium-sized Central American primates characterized by moderate sexual dimorphism [96–99] and moderate seasonal breeding, although births have been documented to occur year round [100]. They reside in groups of approximately 20 individuals, with close to twice as many adult females as males in most groups [101]. Females are philopatric, while males disperse from their natal group around the age of four and continue to transfer between groups every approximately 4 years throughout adulthood [102]. Dominance hierarchies exist in both sexes but, while females have stable linear hierarchies [103], low rates of intragroup male–male aggression/submission make it difficult (if not impossible) to establish linear ranks among subordinate males [73,83]. In addition to the morphological differences observed between alpha and subordinate male white-faced capuchins [73,82,83], there are also behavioural differences, which are conspicuous to a trained observer. The alpha male is the most vigilant group member, the most active individual during group encounters with predators and extragroup individuals and usually very centrally located [104]. Alpha males are also the recipients of the most grooming by adult females. While both alpha and subordinate adult males engage in copulations and are capable of reproduction, alpha males sire the majority (*ca* 80%) of group offspring and engage in more visible, audible and prolonged copulation sequences with stereotyped behaviours [105–107]. Many of the morphological and behavioural traits we associate with alpha male capuchins are probably mediated by their high androgen levels; our data indicate that alpha male capuchins maintain androgen levels year-round that are significantly higher than those of subordinate males [73,94] (see also [85]). Moreover, male dominance status is not fixed, and white-faced capuchin groups undergo alpha male replacements every few years [108]. Outside of these alpha male replacement events, males display low aggression rates towards other group members, unlike in other primate taxa [109].

We conducted our study in Sector Santa Rosa (SSR) of the Área de Conservación Guanacaste (ACG), located in northwest Costa Rica. The white-faced capuchins of SSR have been under near continuous observation since 1983 [110]. Group demographics (births, deaths, immigrations and emigrations), dominance interactions, and other life-history events are continuously tracked in study groups, and temperature and rainfall are recorded year-round. SSR consists of tropical dry forest in various stages of regeneration following intense restoration efforts beginning in the 1970s [110]. The climate at SSR is highly seasonal [111] and consists of a distinct wet season (May through to November), during which near daily rainfall occurs, and a distinct dry season (December through to April), when rains stop, trees lose their leaves and natural sources of drinking water across the forest disappear [93,112]. Previous research has established that daily rainfall is an accurate measure of seasonality at this site [93,113]. Here, we averaged daily rainfall over the 30 days before each sampling date as a metric for seasonality, as in Orkin *et al.* [113]. This metric is a more meaningful indicator of food and water availability than reporting rainfall values on the day of data collection.

We studied all adult males residing in five groups of capuchins in SSR (*n* = 24 males) between January 2022 and June 2024, excluding periods of group instability for each group (i.e. when the dominance rank status of males is unstable). The age of our study subjects ranged from 6.6−28.6 years old (mean ± s.d. = 12.4 ± 3.4 years old). Male ages were known based on observed date of birth, or estimated at first sighting (e.g. immigrant males) based on comparisons with males of known ages. Our team has tracked numerous males from birth to well into their 20s, and these longitudinal observations enable us to very accurately estimate male ages. During the three-years study period, five of the males changed dominance status, three males immigrated to neighbouring groups, five males were added to the dataset (four of them upon reaching adulthood and one as an extragroup adult immigrant male), and eight males went missing. Group composition and sampling effort are detailed in the electronic supplementary material, table S1.

### 2.2. Behavioural data collection

Our team collected a total of 457 h of focal behavioural follows on a total of 24 males (mean ± s.d. = 19.1 ± 12 h per male throughout the study period) during which all instances of UW were recorded. UW behaviour was defined as ‘rubbing one’s own urine on the body using the palm of the hand and/or foot’. Each time a UW event occurred within a focal follow (mean ± s.d. focal follow duration = 10.4 ± 2.4 min), we recorded focal male ID, date and time using the Prim8 Data Logger app on a tablet. Our data collection protocol was designed such that we aimed to equally sample individuals in the morning and afternoon and cycle through all individuals before re-sampling. Focal follows were scheduled in advance and initiated in all possible conditions, even if there was a high chance of aborting the focal (e.g. monkeys are travelling quickly or are high in the canopy), so as not to bias data against difficult viewing conditions. Focal follows were aborted if the focal individual was continuously out of sight for 2 min or more. If a focal follow had to be aborted and the duration of the focal was less than 5 min, the focal data were discarded.

### 2.3. Chemical data collection and analyses

#### 2.3.1. Urine odour sample collection

**Figure 1.**
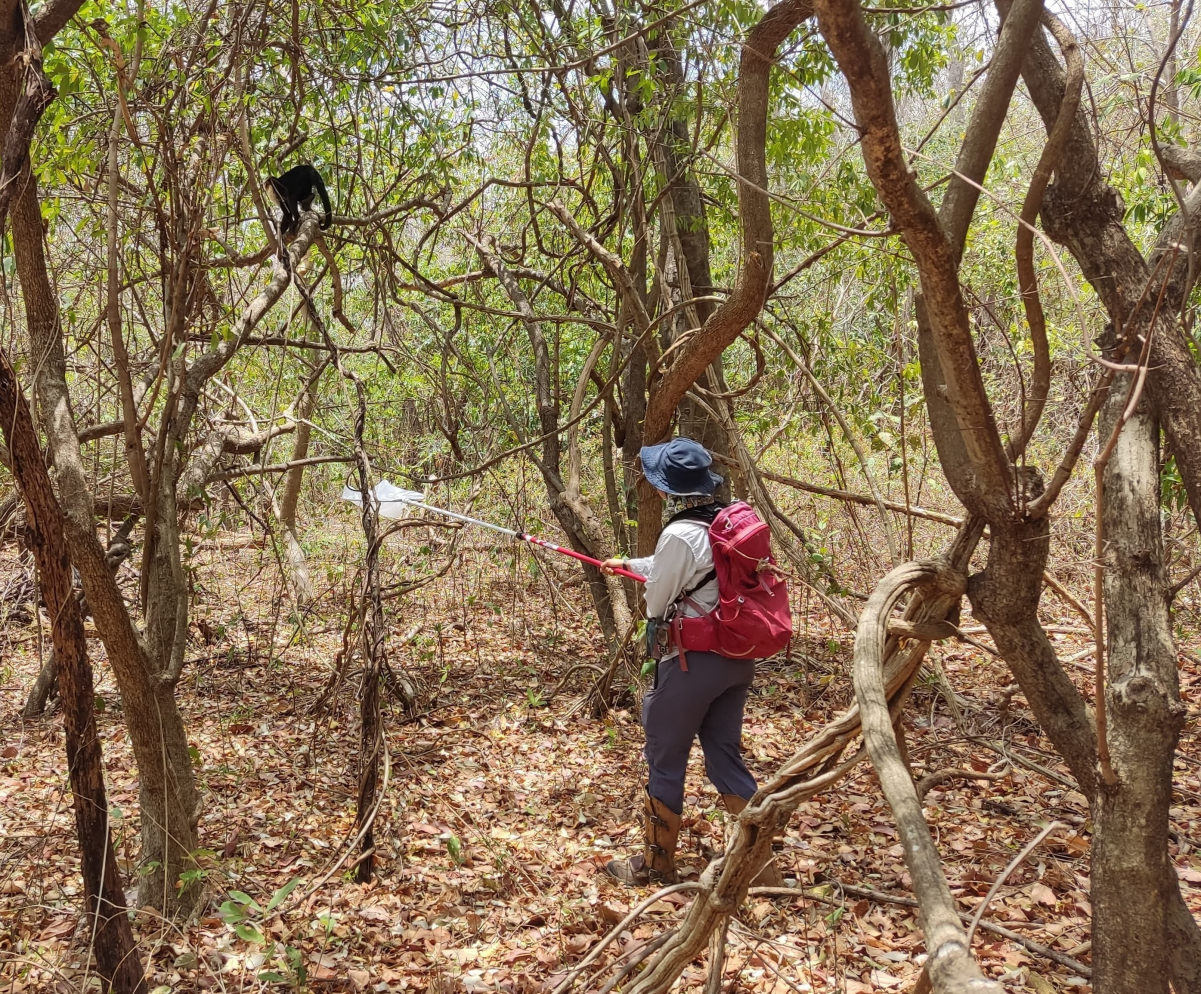
A field researcher holding a telescopic net frame covered with an inert plastic bag to collect urine from a male white-faced capuchin (photo credit B. Cejalvo).

We collected a total of 153 urine samples from 24 males (mean ± s.d. = 6 ± 3 samples per male) between January 2022 and June 2024 (electronic supplementary material, table S1). Excreted urine was collected opportunistically during individual follows of male capuchins, on an inert plastic bag (Kenylon^TM^ oven bags, 46 cm × 70 cm) fixed onto a custom-made telescopic net armature, held underneath the animal (figure 1). Capuchins were habituated to the presence of this equipment in previous years, however, as an added precaution, animals being followed for urine samples were not simultaneously followed for focal behavioural data collection. As soon as urine was collected onto the plastic bag, it was processed for odour extraction *in situ* if the volume collected reached a minimum of 0.1 ml. Several drops of urine (range 0.1−2.0 ml; mean ± s.d. = 0.4 ± 0.5 ml) were pipetted into a 4 ml glass vial using a disposable borosilicate glass Pasteur pipette fitted with a new natural rubber dropped bulb. The urine sample was then incubated for 10 min inside the capped vial on top of a clean sheet of aluminium foil ( ca. 30 cm × 30 cm) laid on the forest floor and shielded from the sun, rain and falling debris, used to create a barrier from the environment below. This incubation step allowed for the build-up of volatiles in the headspace of the vial. To prevent contamination by the human handler, nitrile gloves and face masks were worn while handling the inert bag and while processing the urine. To further reduce potential contamination, all handlers used the same unscented hygiene products and laundry detergent, refrained from using perfumed products, and wore clean, long-sleeve clothing when collecting urine.

Urine odour was sampled into clean stainless steel thermal desorption (TD) tubes (Supelco^TM^) packed with a mixture of absorbent polymers (0.1 g Tenax TA and 0.2 g XAD-2, Sigma-Aldrich, Steinheim, Germany) to cover a wide range of compounds, in particular those of high volatility [114]. After incubating the urine sample in the vial for 10 min, the handler extracted the headspace into a clean TD tube connected by silicone tubing to a portable air pump (SKC Pocket Pump® Touch). This air pump, powered by a removable rechargeable lithium-ion battery, is compact, lightweight and silent, and automatically records the time and volume extracted, as well as the ambient temperature and atmospheric pressure. The pump was set to extract for 2 min at 0.3 l min^−1^, during which the handler steadily held the tube opening directly above the urine sample, at the edge of the vial but without touching the urine. After pumping, the tube was capped tightly with a Swagelok^TM^ cap, wrapped in clean aluminium foil to prevent the cap from unscrewing during transportation, and placed in a sealed individual bag labelled with information on tube ID, animal and group ID, date and time and sample handler ID.

We additionally recorded information regarding the sampling conditions, as these may have an effect on the chemical composition of the sample. First, we recorded if the urine sample was collected by direct catch (i.e. collected on the inert bag directly from the urinating animal) or indirect catch (i.e. collected on the bag after it fell through vegetation). We also recorded the urine volume sampled (in ml), as well as the ambient temperature at the time of sampling (in °C). Finally, we measured the specific gravity (SPG) of the urine sample by pipetting three drops of urine onto the prism of a hand-held digital refractometer (MISCO®), which enabled us to control for sample concentration in our analyses. We took two SPG readings per urine sample and used the mean SPG value as a variable. Highly concentrated samples, for which the SPG reading was out of range, were assigned the maximum value of 1.065, as indicated by the manufacturer. Control samples of air—including the full procedure with bags, pipettes and vials used to collect urine—but without capuchin urine, were collected once per sampling day (i.e. ‘air controls’, *n* = 50). In addition, unopened TD tubes served as blanks to control for compounds originating from the sampling material and not the sampling environment *per se* (i.e. ‘TD tube controls’, *n* = 6). Upon return to the field station, the sample TD tubes were stored at ambient temperature until shipping to Germany for chemical analysis at Leipzig University. The average storage time for samples was mean ± s.d. = 126 ± 53 days, shipped and analysed in 11 batches. All samples of a batch were run within one week.

#### 2.3.2. Gas chromatography-mass spectrometry analyses

Chemical analyses were carried out by TD GC-MS following established methods for the analysis of TD tube samples [114,115] using a Shimadzu TQ8040 GC-MS coupled to a TD unit (TD-20, Shimadzu). The analytical parameters used are detailed in the electronic supplementary material, table S2 and an example of male capuchin urine chemical profiles is shown in the electronic supplementary material, figure S1. An unused clean TD tube was run every 10 samples, and an empty clean TD tube (i.e. without any absorbent) at the start and at the end of every batch of samples, to control for contamination from the instrument or the laboratory environment (i.e. ‘laboratory controls’). The trapped organic compounds were desorbed from the tube into the GC-MS and any of the remaining residues were removed by cleaning the tubes with heat under a constant flow of nitrogen in a thermal conditioner (Clean-Tube, Scientific Instruments Manufacturer SIM, Oberhausen, Germany), allowing tubes to be sent back to the field to be reused.

GC-MS data were processed following an established, semi-automated procedure [114,115]. In brief, a preliminary peak list was created through automated peak detection and alignment by retention time (RT) in the software AMDIS 2.73 [116] followed by manual correction of peak assignment by considering mass-to-charge (*m/z*) ratios of peaks in addition to RTs. At this stage, the peak list contained individual peak IDs characterized by their mean RT, mean area and principal *m/z* ratios, without attempting compound identification. Batch-related shifts in RT were taken into account during peak assignment to ensure that RTs in different batches corresponded to the same compound. Specifically, all peak RTs were corrected based on a regression equation built from the individual RTs from an *n*-alkane reference mixture run at the start and at the end of the project, as well as a number of common and readily identifiable silane contaminants recovered from most samples. The resulting peak list was used as a custom peak library, which was applied to the chemical profiles in the software GC-MS Solutions v.4.20 (Shimadzu, Kyoto, Japan) to extract the peak areas of all specified peaks within their assigned RT range (i.e. within ± 50% of the library range entry for each peak) and a signal-to-noise ratio threshold ≥1. This resulted in more accurate detection and integration of the library peaks than entirely automated procedures, and was more time-efficient and repeatable than fully manual profiling. The complete peak list was further refined through manual steps in R v.4.5.0 [117] to exclude peaks present in more than 10% of the laboratory controls and peaks found in higher amounts in the field or laboratory controls than in the urine samples (i.e. indicative of a spurious origin), peaks found exclusively in a single batch of samples (i.e. suggesting they are contaminants from the analytical system used rather than originating from the animals themselves), as well as rare peaks (i.e. present in fewer than three samples), resulting in a final list of 88 compounds of interest (electronic supplementary material, table S3). In addition, we visually determined the noise threshold as a peak area of 1000, as peaks below this value were not reliably separable from background noise, and manually set all peak areas below this threshold to 0. Finally, we tentatively identified compounds using the National Institute of Standards and Technology mass spectral library (NIST14) on the basis of their mass spectra and retention indices, which were calculated based on an *n*-alkane reference mixture analysed under identical conditions. As common in compound profiling studies, verifying the identity proposals provided by the NIST mass spectral library was limited, since for many of the compounds authentic standards are not available. When high-quality compound identification was not given in NIST (i.e. identity match < 75%), we made an attempt to identify its main chemical characteristics and provided an approximate compound name.

Before the statistical analyses, we further calculated relative peak abundances (RPA) = peak area/sum of peak areas in sample × 100 (excluding contaminants) and applied either a log (*x* + 1) transformation or a more complex arcsine-log transformation (i.e. log(asin(√(RPA/100)) + 0.01)). The log (*x* + 1) transformation was used to centre the data distribution and to reduce the relative impact of abundant compounds while maintaining relative differences in the abundance of compounds within a sample, and the arcsine transformation was applied to reduce bottom-ceiling effects when modelling the data as a Gaussian response variable [115,118–121].

### 2.4. Statistical analyses

#### 2.4.1. Prediction (i): alpha males perform urine washing more frequently than subordinate males

To test prediction (i), we applied a generalized linear mixed model (GLMM) approach. We used the number of UW a given male performed within each respective focal follow as our unit of analysis (i.e. count data, mean ± s.d. = 0.2±0.5 occurrences per focal follow). We fitted a GLMM adapted to zero-inflation (glmmTMB function in R package ‘glmmTMB’ v.1.1.11 with argument ziformula = approx. 1 [122]), with a Poisson family and log link function suitable to count data. We used UW rates data (i.e. count per focal follow) as the response variable, and fitted the predictors male dominance rank status (two levels, alpha versus subordinate), age (continuous in years), group ID (five levels) and seasonality (i.e. daily rainfall averaged over the 30 days before each date of our study period, continuous in mm). We also included the interaction between dominance rank status and seasonality, since the social stability of the capuchin groups has previously been shown to be impacted by seasonality in this population [93–95]. Age and seasonality were *z*-transformed to facilitate model convergence and interpretation [123]. In addition, we included male ID (24 levels), date (252 levels) and observer ID (15 levels) as random effects to control for repeated measures across individuals, days and observers, respectively. Finally, we added log-transformed focal follow duration (in minutes) as an offset in our model to account for variation in the duration of focal follows. We tested the overall significance of fixed effect predictors by comparing the full model with a null model lacking the predictor of interest using a likelihood ratio test (LRT; ANOVA function with *χ*^2^ test in R package ‘stats’). We then tested the significance of individual predictors on the rate of UW using the ANOVA function in R package ‘car’ v.3.1−3 [124]. Inspection of the full and null model residuals (R packages ‘predictmeans’ v.1.1.1 [125]; and ‘DHARMa’ v.0.4.7 [126]) did not reveal any obvious deviation from assumptions for this type of linear model. We further plotted the interaction between rank status and seasonality from our full model using the plot_model function in R package ‘sjPlot’ v.2.8.17 [127].

#### 2.4.2. Prediction (ii): the urinary chemical profiles of alpha males are distinct from those of subordinate males

To test prediction (ii), we used a combination of two different statistical approaches. As chemical profiles represent high-dimensional data, we first used a multivariate approach that allows testing entire chemical profiles rather than single compounds of the profile as the effective unit of analysis (prediction iia). We compared pairwise similarities of olfactory profiles with a permutational multivariate analysis of variance (PerMANOVA; adonis2 function in R package ‘vegan’ v.2.6−10 [128], using 999 permutations and the Bray–Curtis dissimilarity index [129]. We used log (*x* + 1)-transformed RPA values of each sample (*n* = 153) and compound (*n* = 88) as a response matrix. We included male dominance rank status and seasonality, as well as age and group ID, as individual predictors. To be conservative in our analyses, we additionally included control predictors regarding the sampling and analysis conditions, as these may have an effect on the chemical composition of the sample: urine catch type (two levels: direct or indirect catch), mean sample SPG (continuous), volume sampled (continuous), ambient temperature at the time of sampling (continuous), handler ID (17 levels) and GC-MS batch (11 levels). We further used collection date as a continuous variable to control for potential methodological issues (i.e. to assess if there was a linear change in the abundance of particular compounds over time). We *z*-transformed the continuous variables to facilitate model convergence and interpretation. We controlled for repeated samples of the same individuals by including male ID as stratum (similar to a random effect in a linear model approach). We first assessed the overall significance of all predictors of the model (argument ‘by = NULL’ in adonis2 function), after which we looked at the marginal effects of each predictor (argument ‘by = ‘margin’’). To be consistent with our behavioural analyses, we initially included in our model the interaction term between rank status and seasonality. This interaction term was non-significant in the marginal PerMANOVA result (*R*^2^ = 0.003, *F*_1,152_ = 0.641, *p* = 0.762) and was omitted from subsequent analysis. We tested the assumption of homogeneity of multivariate variance (permutest function in ‘vegan’, using 999 permutations) on the measured multivariate homogeneity of variance for each level of categorical predictor (betadisper function in ‘vegan’). This assumption was verified for our main predictor of interest, rank status. The assumption was not met for group ID or for the control predictors male ID, handler ID and GC-MS batch, but since a violation of homogeneity of variance may only lead to false positive results for these particular predictors, we did not consider this to be a consequential issue for our hypothesis testing.

We then investigated the influence of test predictors on sample chemical composition at the level of the compound, while taking into account multiple fixed and random effects of predictors (prediction iib). We implemented a random slopes model approach following Jamil *et al.* [130], which uses a vectorized multivariate data matrix and includes the matrix rows (i.e. samples) and columns (i.e. compounds) as random factors. We fitted linear mixed models (LMMs, lmer function in R package ‘lme4’ v.1.1−37 [131]) with arcsine-log (*x* + 1)-transformed RPA value of each sample and compound as response (*n* = 2557 RPA values). We included the test predictors male dominance rank status, seasonality, age and group ID, as well as the control predictors catch type, mean SPG, urine volume, ambient temperature and collection date (as a continuous variable), as fixed effects in our LMMs. In addition, sample ID, compound ID, male ID, handler ID, collection date (categorical, 94 levels) and GC-MS batch were fitted as random effects. All continuous variables were *z*-transformed. An important aspect for model formulation and interpretation in this random slopes approach is that we did not expect any predictor to affect all compounds in the same manner, especially since in our data the compound RPAs were adjusted to the same ratio per sample. Consequently, the effects of interest in this model were not the fixed effects of individual predictors, but the interaction between predictors and compound IDs (first described by Weiß *et al.* [115]). As compound ID was a random effect in the model, this interaction between predictor and compound ID corresponded to the random slope of predictor within compound ID. We therefore fitted the random slopes of all fixed effects predictors within compound ID. We also included the random slopes of dominance rank status and catch type within batch, as well as catch type within handler ID and within male ID, to achieve more reliable *p*-values. None of the other fixed effect predictors varied sufficiently within the random effect levels to model their random slopes [132]. The full and null models used in this approach fulfilled the assumption of normal residuals and homoscedasticity, yet an inspection of residuals against fitted values indicated some autocorrelation (see prediction (i) above for methods). However, as the models showed no indications of collinearity (largest Variance Impact Factor = 2.4 for group ID; R package ‘car’), and the observed heterogeneity could be explained by the overall distribution of our data, we did not consider this problematic.

## 3. Results

### 3.1. Prediction (i): alpha males urine washing more frequently than subordinate males

**Figure 2.**
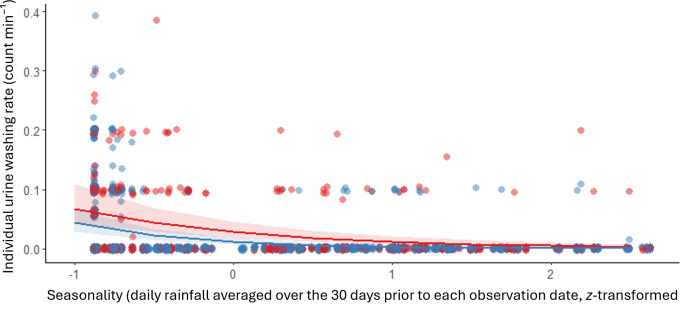
Individual urine washing rates (i.e. counts per minute) across seasonality values (i.e. daily rainfall averaged over the 30 days before each observation date) for alpha (red) and subordinate (blue) male white-faced capuchins. Raw data points (colour dots) are plotted, as well as the linear model estimates (i.e. predicted UW rates; coloured lines) and associated standard error (shaded ribbons) from the full generalized linear mixed model. Seasonality was *z*-transformed to facilitate model convergence, where lower values indicate drier conditions (range of non-transformed values = 0−17 mm; mean ± s.d. = 4.3 ± 4.9).

We recorded 522 UW events observed during 457 h of focal follows conducted on all 24 adult males in our study. The individual UW rate per minute was 0.02 ± 0.01 (mean ± s.d.) UW events min^−1^ (alpha males: 0.03 ± 0.01 UW events min^–1^; subordinate males: 0.02 ± 0.01 UW events min^–1^; electronic supplementary material, table S1). We found variation in the frequency of UW behaviour in our study population (full–null model comparison using LRT: $\chi_{8}^{2}$ = 132.730, *p* < 0.001). The interaction between dominance rank status and seasonality was significant (table 1). Specifically, alpha males engaged significantly more frequently in UW than subordinate males, and the drier the weather (i.e. less rainfall), the more UW alpha male capuchins performed (table 1; figure 2). However, age and group ID were not significant predictors of UW behaviour (table 1).

**Table 1.** Results of a generalized linear mixed model adapted to zero-inflation assessing differences in the frequency of urine washing in male capuchins. (Significant *p*-values are indicated in bold.)

fixed predictors	*χ* ^2^	d.f.	*p*
male dominance rank × seasonality	6.201	1	**0.013**
male dominance rank	13.413	1	n/a^a^
seasonality	99.551	1	n/a^a^
male age	0.557	1	0.456
group ID	5.928	4	0.205

^a^ Not presented since these terms are comprised in the significant interaction and thus having a very limited interpretation.

### 3.2. Prediction (ii): the urinary chemical profiles of alpha males are distinct from those of subordinate males

We detected a total of 88 unique volatile compounds from the 153 urine samples collected from 24 male capuchins (mean ± s.d. = 17 ± 6 compounds per individual profile). We tested the effect of male dominance rank on the variation in chemical composition of urine using a combination of statistical approaches. First, a PERMANOVA established that the urine chemical profiles collected showed significant pairwise variation (full model using the ‘null’ argument: *R*^2^ = 0.530, *F*_38,152_ = 3.385, *p* = 0.001). When looking at the marginal effects of each predictor on pairs of samples, we found a modest yet significant effect of male dominance rank status on pairwise (dis)similarity across urinary profiles, where samples from the same dominance category were significantly more similar than those from different categories (table 2). Of the other variables tested, GC-MS batch was the only other significant predictor of differences observed across samples (table 2).

**Table 2.** Results of a permutational multivariate analysis of variance (Adonis) assessing the variation of urine chemical composition across pairs of samples. (Male ID was used as stratum to account for repeated samples from the same males. Significant *p*-values are indicated in bold.)

model predictor	*F*	d.f.	*R* ^2^	*p*
male dominance rank	2.384	1	0.010	**0.046**
seasonality	0.981	1	0.004	0.468
male age	0.524	1	0.002	1.000
group ID	1.307	4	0.022	0.596
catch type	0.852	1	0.004	0.507
mean SPG	1.452	1	0.006	0.168
sample volume	1.438	1	0.006	0.265
ambient temperature	1.016	1	0.004	0.563
handler ID	1.244	16	0.082	0.070
GC-MS batch	2.863	10	0.118	**0.001**
collection date (continuous)	1.337	1	0.006	0.132
residuals		114	0.470	
total		152	1.000	

Our second approach investigated the influence of test predictors on sample chemical composition at the level of the compound using random slopes models. The full–null model comparison showed that the composition of odour profiles was not affected by the combination of test predictors (LRT: $\chi_{4}^{2}$ = 5.920, *p* = 0.205), thus no attempt was made at testing the effect of individual test predictors.

Finally, we were able to provide a tentative identity to 45% of the 88 compounds retrieved from capuchin urine samples (electronic supplementary material, table S3). The majority of the compounds identified were hydrocarbons (i.e. alkanes, cycloalkanes and aromatic hydrocarbons; 12.5%), alcohols (5.7%), ketones (4.5%), esters (4.5%) and carboxylic acids (2.3%). Many compounds contained a nitrogen element (e.g. amines, amides, pyrazines; 21.6%). Over 14% of the compounds identified contained an aromatic group (i.e. phenol or furan); the rest were aliphatic (i.e. straight, branched or cyclic compounds; 34.1%).

## 4. Discussion

In this study, we combined analyses of behavioural and chemical data to assess the potential for urine to act as signalling medium in dominance-based olfactory communication by male white-faced capuchin monkeys. Specifically, we first examined UW behaviour, which is common in this species, as a potential mechanism for facilitating olfactory signalling. We found that alpha males engaged in more UW events than subordinates, significantly so in the dry season, which aligns with previous findings on this species [68,77]. This pattern was not driven by male age nor group. We also found an effect of male dominance rank on the chemical dissimilarity between the 153 urinary profiles analysed, where profiles from male capuchins with similar dominance ranks were significantly more similar than profiles of males from a different dominance category.

### 4.1. Prediction (i): alpha males urine washing more frequently than subordinate males

Our results showed that the relationship between male dominance and UW behaviour is more pronounced as the conditions in SSR become drier, bolstering the communicative potential of this behaviour. Studies of the closely related tufted capuchins (*Sapajus apella*) have also observed higher UW rates in drier, hotter weather, leading researchers to suggest that UW may have a main function in mobility, by improving grip on substrates in the environment or in thermoregulation [66,77–79]. However, if these were the primary roles for UW behaviour in this taxon, then all individuals should be engaging in UW at relatively similar rates as all would reap these potential benefits. Not only would any potential urinary signals be more enduring in the dry season in comparison with the wet season when they would be washed away, but the dry season is also characterized by increased intergroup encounters in this population as multiple groups are forced to share scarce water sources [83,133]. These encounters involve the exchange of low to high grade aggression between males from different groups, sometimes leading to severe injuries and even death, particularly among alpha males who are the primary participants [103,134].

While Campos & Fedigan [77] found no relationship between capuchin male UW frequency and location in the home range, including in and around water sources, it may be that the sheer volume of scent marks deposited by alpha males across their home range serve to advertise their quality or dominance status and reduce costly intergroup conflict [135,136]. For instance, urine chemical composition has been shown to reflect an individual’s foraging ability, considered an honest indicator of the individual’s quality as mate. In an experimental study, Ferkin *et al.* [53] showed that female meadow voles (*Microtus pennsylvanicus*) preferred the urine of males fed on a high-protein diet over those fed on a low-protein diet. A similar function has been proposed for the urine-based scent-marking behaviour by male house mice (*Mus domesticus*), in which urinary scent marks deposited by males have been shown to broadcast reliable information about individual male competitive ability and function in territorial marking and defence [54,136]. Capuchins engage in a multitude of olfactory-related behaviours that include sniffing and licking of substrates and the Flehmen response. We have observed many incidences of male capuchin sniffing trees occupied by group members or by extragroup conspecifics. For example, K. Jack (unpublished data, 2021) observed a lone male spend nearly 10 min sniffing the branches of a tree that she observed a neighbouring group to rest on the previous day. To advance our understanding of the potential of urine to act as a chemical messenger in this species, field experiments need to be conducted to observe the responses of individuals when they encounter the urinary odours of alpha and subordinate males.

### 4.2. Prediction (ii): the urinary chemical profiles of alpha males are distinct from those of subordinate males

We found some evidence that the overall urinary chemical profiles of alpha male capuchins are significantly different than that of subordinate males. We uncovered a total of 88 unique compounds from the 153 male capuchin urine volatile profiles analysed, with an average of 17 compounds per individual profile. This was within the range of previous findings reported from other mammals. For instance, 74 compounds were retrieved from the urine of strepsirrhines [39], 55 from white-tailed deer [48], 55 from lions, *Panthera leo* [137], 47 from brown rats, *Rattus norvegicus* [138], and 30 from ferrets, *Mustela furo* [45]. The combination of TD tubes as a sensitive volatile collection method routinely used for plant and animal chemical communication, and a two-dimensional GC system with thermal desorber as an efficient compound separation technique, may be partially responsible for the relatively high number of compounds retrieved from the samples. The urinary compounds tentatively identified in this study were mainly hydrocarbons, ketones, esters, alcohols and carboxylic acids, sometimes containing an aromatic group, many of which have been reported in other primates and other mammalian taxa, mainly carnivores, rodents and artiodactyls [2,3,27,139]. A number of these compounds could potentially play a role in the chemical communication of dominance rank status in white-faced capuchins.

We found no significant effect of male dominance rank, age or group ID, nor seasonality, on individual compound variation. Studies on other mammalian taxa have evidenced chemical variation in urine volatile composition in association with male dominance rank status (e.g. in rodents [87,140,141]; and deer [48]), age (e.g. in rodents [47]), and group membership (e.g. in lions [137]). It is possible that differences in urine volatile composition at the level of the compound were masked by unobserved environmental and methodological variables. Indeed, our finding that the control predictor GC-MS batch, i.e. the group of TD tubes analysed together in a single run on the GC-MS instrument, was a significant predictor of urine chemical composition in our model, indicates that methodological biases are responsible for part of the variation observed in our chemical data. Our GC-MS measurements spanned a 2.5 year period and unavoidably involved many individual batches, with several other GC-MS runs from other projects in between, which led to the batch effect detected. The limited number of TD tubes we had available for this study (owing to their high cost and the number of controls we included) meant sending tubes back and forth between the field site and the University of Leipzig for GC-MS analysis so that we could keep the sampling pipeline going throughout the entire study period. To adjust for this batch effect our chemical analyses pipeline included a manual correction of peak assignment with samples from all batches taken together. This step permitted us to reduce the batch effect in our samples, albeit not entirely, as GC-MS batch still explained 12% of the variance found in our PERMANOVA model. Such variation across analysis batches is a commonly reported issue in GC-MS [142]. Aware of this limitation, we organized our sample transportation and analysis to limit the number of batches as much as possible, and we accounted for these methodological sources of variation in our statistical analyses by including control variables such as batch and date either as random or as fixed effects in our models.

### 4.3. Limitations

As with any study of chemical ecology in wild animals, our research has some key limitations. In the chemical profiling of animal odours, and notably urine, it is often difficult to determine which proportion of the volatiles composing chemical profiles are produced by the animals, versus present in the environment, introduced through diet, or produced by commensal bacteria [2,139,143]. For instance, β-pinene is almost certainly of non-mammalian origin, because its metabolic pathway only exists in plants, fungi, and bacteria [139]. This compound may originate from diet, and is liberated in urine as an unmetabolized compound. DelBarco-Trillo *et al.* [144], who compared the chemical composition of bladder and voided urine from aye-ayes (*Daubentonia madagascariensis*), reported that 17% of the compounds retrieved were found uniquely in the excreted urine. This suggests a change in urine composition as it travels through the urogenital tract, and putative chemical reactions prompted by contact with the environment and with bacteria, which may have occurred during our sampling. Furthermore, our choice of odour collection methodology, i.e. extraction of the headspace above urine into a TD tube, samples primarily volatile compounds, and some semi-volatile ones, probably missing some semi- and non-volatile compounds in urine [114]. In addition, urinary odour characteristics may develop over time after deposition as the urine constituents degrade and transform [1,6]. Moreover, as is common in field studies spanning a long time period, our samples had to be stored in between each shipment to the GC-MS laboratory. This storage time varied across samples and across batches, and although TD tubes are amongst the most stable scent traps available for field sampling [145,146], prolonged storage and shipment at room temperature could have led to some degradation of the odour samples [147,148].

### 4.4. Summary and future directions

We investigated the chemical composition of capuchin urine, and combined urinary odour profiling with behavioural observations of UW. We found that alpha males engaged in more UW than subordinate males, significantly so in the dry season. We also found a modest effect of male dominance rank on overall urine sample chemical dissimilarity. Given the limited influence of male dominance rank, as well as the demographic and ecological predictors tested in explaining variation in sample chemical composition, our findings indicate potentially complex ecological drivers of urinary odour variation in white-faced capuchins. Fruitful future studies may include exploring the variation in UW and urinary profiles in relation to periods of group instability and in the context of alpha male replacements. To advance our understanding of the potential of urine to act as a chemical messenger in this species, field experiments are needed to observe the responses of individuals when and where they encounter the urinary odours of both intra- and extragroup alpha and subordinate males. This future research will help shed additional light on possible functions of UW behaviour, particularly in the context of male dominance rank status, and further the understanding of primate chemical communication.

Another future direction includes investigating the links between the whole chemical characteristics of urine (i.e. across a range of volatility levels) and the animal’s sensory abilities. For instance, many animals display a number of physiological and behavioural adaptations for detecting and processing chemicals of low volatility [4]. Many mammals, including platyrrhine primates, possess a functional vomeronasal organ, located above the palate inside the mouth, specialized in the detection of chemicals of low volatility [149]. A recent assembly and high-coverage analysis of the *C. imitator* genome by Orkin *et al.* [150] found seven intact vomeronasal receptor genes that appear to be under purifying selection, indicative of a functional role in social communication. An exciting future direction could be to further investigate odourants of low volatility as well as vomeronasal receptor sensitivity for specific compounds of interest in this species. Additional research integrating behavioural, chemical and genetic studies are needed to better understand how olfaction contributes to social communication and drives phenotypic variation in primates, a highly diverse order of mammals.

## Data Availability

The data and R code supporting this article are available on the Dryad repository [153]. Supplementary material is available online [154].
